# Loss of core-fucosylation of SPARC impairs collagen binding and contributes to COPD

**DOI:** 10.1007/s00018-022-04381-4

**Published:** 2022-06-07

**Authors:** Tsai-Jung Wu, Sheng-Hung Wang, Eric Sheng-Wen Chen, Hsiu-Hui Tsai, Yi-Chieh Chang, Yi-Hsin Tseng, John Yu

**Affiliations:** 1 Institute of Stem Cell and Translational Cancer Research, Chang Gung Memorial Hospital at Linkou, Taoyuan, 333011 Taiwan; 2 Institute of Cellular and Organismic Biology, Academia Sinica, Taipei, 11529 Taiwan

**Keywords:** COPD, Fut8, Core-fucosylation, SPARC, Matricellular protein, Collagen

## Abstract

Chronic obstructive pulmonary disease (COPD) is a progressive lung disease with high morbidity and mortality worldwide. Although several mechanisms to account for deleterious immune effects were proposed, molecular description for the underlying alveolar structural alterations for COPD is lacking. Here, silencing of α1,6-fucosyltransferase (Fut8), the enzyme for core-fucosylation and highly expressed in lung stem cells, resulted in alveolar structural changes in lung organoids, recapitulating COPD. Site-specific mass spectrometry analysis demonstrated that the secreted protein acidic and rich in cysteine (SPARC), which binds collagen, contains a core-fucosylation site in its VCSNDN^cf^K glycopeptide. Biacore assay showed markedly reduced collagen binding of SPARC lacking core fucosylation. Molecular dynamics analysis revealed that core fucosylation of SPARC-induced dynamic conformational changes in its N-glycan, allowing terminal galactose and N-acetylglucosamine to interact with K150, P261 and H264 residues, thereby promoting collagen binding. Site-specific mutagenesis of these residues also resulted in low affinity for collagen binding. Moreover, loss of collagen and decline of core fucosylation were observed in COPD lung tissues. These findings provide a new mechanistic insight into the role of core fucosylation of SPARC in cell–matrix communication and contribution to the abnormal alveolar structures in COPD.

**Supplementary Information:**

The online version contains supplementary material available at 10.1007/s00018-022-04381-4.

## Introduction

Chronic obstructive pulmonary disease (COPD), including emphysema and chronic bronchitis, is a progressive disease with high morbidity and mortality worldwide. The cause of COPD has been linked to chronic exposure to lung irritants, such as cigarette smoke or other air pollutants, which can lead to abnormal inflammation and airflow limitation. In addition, the structural remodeling refers to alterations of the extracellular matrix (ECM) in the airway and parenchymal compartments in patients with COPD [1]. The enlarged air spaces with emphysematous destruction of alveolar walls and loss of gas exchange surface caused by ECM degradation are hallmarks of COPD. Various hypotheses to account for COPD progression have been proposed, including oxidative stress, protease imbalance, airway remodeling etc.; however, the detailed pathobiological mechanisms remain unclear.

Protein glycosylation is the most common but least understood post-translational modification. The carbohydrates on the cell surface play important roles in many physiological and pathological events. Fucosylation, in which fucose from guanosine diphosphate L-fucose (GDP-fucose) is transferred to glycoproteins or glycolipids, is catalyzed by fucosyltransferases (Futs). In mammals, α1, 6-fucosyltransferase (Fut8) is responsible for core-fucosylation, where fucose was added to the asparagine-linked N-acetylglucosamine (GlcNAc) in α1, 6-linkage in the core structure of N-glycan. In Fut8^−/−^ mice and cigarette smoke-exposed Fut8^+/−^ mice, core-fucosylation has been implicated in the development of emphysema [2, 3]. Consistent with these findings in mice, pathogenic variants of *FUT8*, which resulted in congenital disorders of glycosylation with defective core-fucosylation, showed growth retardation and respiratory complications [4, 5]. One autopsy report implied an association of the Thr267Lys polymorphism of Fut8 gene with pulmonary emphysema [6]. In addition, COPD patients with lowered Fut8 activity in serum experienced exacerbations more frequently [7]. On the other hand, N-glycan analysis of the plasma in 1914 individuals showed that core-fucosylation was decreased in smokers [8]. Recently, we also found that core-fucosylation of lung glycoproteins was altered by chemicals or environmental risk factors which led to lung disorders [9]. Therefore, even though Fut8 may not genetically be impaired, the core-fucosylation of the Fut8 target proteins might be relevant to the development of COPD.

Fut8^−/−^ mice display multiple organ dysfunctions and a high mortality rate soon after birth [2]. Given limitations for mechanistic studies using this Fut8^−/−^ mice, we developed a defined organoids culture from isolated lung stem cells (LSCs) [10, 11] to examine whether diminished core-fucosylation could recapitulate the structural changes observed in alveolar sacs in COPD. In addition, we performed the site-specific quantitative mass spectrometry (MS) proteomic platform to identify the core-fucosylated proteins in LSC after Fut8 knockdown. Based on these analyses, the matricellular protein, secreted protein acidic and rich in cysteine (SPARC) was identified as a Fut8 target protein and validated that alteration of core-fucosylation affect its function. Afterwards, Biacore analysis and molecular dynamics simulation were employed to assess the conformational alterations of SPARC and its interactions with the collagen in ECM. Finally, structural mutations generated through in situ mutagenesis validated the detailed structural interactions between SPARC and collagen. Therefore, in addition to the conventional role of inflammatory cells in the pathogenesis for COPD, our findings provide a new mechanistic insight into the critical involvement of core fucosylation of SPARC in cell–matrix communication and contribution to the abnormal alveolar structures in COPD.

## Materials and methods

### Clinical specimens

For validation of collagen expression in human lung tissue, the formalin-fixed, paraffin-embedded adjacent normal lung tissues from patients with lung cancer were obtained from the Tissue Bank of Chang Gung Memorial Hospital, Taiwan. Eligible subjects were selected and divided into two groups: one with signs of COPD (airflow obstruction, *n* = 8) and the other with lung function within the normal range (normal, *n* = 6). Paraffin blocks were sectioned and stained using a Masson trichrome stain kit (Sigma, HT15), according to the manufacturer’s instructions. Masson Trichrome stains collagen fibers in blue and muscular fibers in bright red. Sections were examined by pathologists and digital images were captured with an Aperio Scope AT Turbo Slide Scanner (Leica Biosystems) at 40 × magnification. The amount of collagen deposited was quantified using the software StrataQuest (TissueGnostics GmbH) and is presented as a percentage of lung section surface area.

### Antibodies

For immunostaining, the following antibodies were used: Oct-4 (Santa Cruz; sc-5279), proSP-C (Sigma-Aldrich; AB3786), AQP5 (Sigma-Aldrich; AB15858), E-cadherin (BD Bioscience; 610182), collagen type I (Sigma-Aldrich; AB765P), phycoerythrin (PE)-conjugated anti-CD157 (BioLegend, 140203), FITC-conjugated anti-CD54 (BD Bioscience, 561898), and allophycocyanin (APC)-conjugated anti-CD45 (BioLegend, 103111). For Western blot and immunoprecipitations, the following antibodies were used: SPARC (Cell Signaling Technology; #5420, #8725), collagen type I (Sigma-Aldrich; AB765P; Santa Cruz, sc-59772), and a β-actin (Sigma-Aldrich; A5441). Secondary antibodies conjugated to HRP were purchased from Jackson ImmunoResearch. Secondary antibodies conjugated to Alexa Fluor 488, Alexa Fluor 555, Alexa Fluor 647 were purchased from Life Technologies.

### Cell culture

Neonatal CD1 (ICR) mice (between days 1–3 after birth) (BioLascoice Taiwan Co., Ltd) were used to prepare LSCs. Isolation and primary culture of LSCs was performed as previously described [10, 12]. Briefly, the lungs were diced and incubated with 0.1% protease type-XIV (Sigma, P5147) and 10 μg/ml DNase I (BioShop, DRB001) in DMEM at 4 °C for 16–20 h. The reaction was quenched with an equal amount of medium containing 10% FBS and filtered through a 100 μm cell strainer to remove the cell debris. The cell pellet was resuspended in RBC lysis buffer (150 mM NH_4_Cl, 10 mM KHCO_3_, and 0.1 mM EDTA), incubated for 2 min and centrifuged at 300×*g* for 5 min at 4 °C. The cell pellet was resuspended and stained with PE-conjugated anti-CD157, FITC-conjugated anti-CD54 and allophycocyanin (APC)-conjugated anti-CD45 antibodies. Fluorescence-activated cell sorting was performed using a FACSAria™ III cell sorter (BD Bioscience). For culture, we used isolated CD45^−^CD54^+^CD157^+^ lung cells that were maintained in DMEM medium supplemented with 10% FBS, 1% insulin–transferrin–selenium (ITS) (Thermo Fisher Scientific, 41400) and 1 ng/ml EGF (Thermo Fisher Scientific, PHG0314) on a collagen I-coated plate. To induce cell differentiation, attached LSCs were incubated in MCDB-201 medium (Sigma, M6770) supplemented with 1% FBS, 1% ITS and 10 ng/ml EGF for 7 or 14 days to become type II alveolar epithelial cells (AECII) or type I alveolar epithelial cells (AECI), respectively. Organotypic culture of LSCs was achieved with the use of a combination of cell culture techniques, which were modifications of the method described by Lee et al. [13]. HUVEC cells were cultured using an Endothelial Cell Growth Medium-2 Bullet Kit (EGM-2) (Lonza, CC-3162) on gelatin-coated plates. For 3D organotypic culture experiments, HUVEC cells were grown up to 90% confluence one day prior to co-culture in 8-well chamber slides. On the next day, medium was removed, 100 μl of growth factor reduced Matrigel (diluted 1:1 in DMEM/F12 medium, Corning, 354230) in its liquid state was added to cover the endothelial cells and allowed to gelatinize at 37 °C for 30 min prior to LSC plating. Afterwards, LSCs in EGM-2 growth medium were seeded on the top of the Matrigel at 1 × 10^4^ cells/cm^2^. The culture media were refreshed every two days. The control HEK-293T (293T-WT) and FUT8-knockout HEK-293T (293T-Fut8KO) cells were kindly provided by Dr. R-B Yang [14] for the expression of SPARC proteins with or without core-fucosylation. These cells were cultured in DMEM medium supplemented with 10% FBS.

### Measurement of Fut8 activity

The enzymatic activity of Fut8 was determined as described previously [15]. Briefly, a 5 μg sample of cell lysate was mixed with the assay buffer (200 mM MES, pH 7.0, 1% Triton X-100, 500 μM GDP-L-fucose, and 50 μM GnGn-bi-Asn-(4-(2-pyridylamino)butylamine) (GnGn-Asn-PAPB). After incubation at 37 °C for 2 h, the reaction was stopped by boiling. The sample was then centrifuged at 15,000×*g* for 10 min, and 10 μl aliquot of the supernatant was subjected to HPLC with a fluorescence detector (Ex. 310 nm and Em. 380 nm). The activity of Fut8 is expressed as pmol of GDP-fucose transferred to the acceptor GnGn-Asn-PABA per hour per milligram of protein.

### Small interfering RNAs (siRNAs) transfection

In knockdown experiments, cells were transfected with siRNA oligonucleotides using Lipofectamine RNAiMAX (Invitrogen, 13778150) according to the manufacturer’s instructions. Stealth RNAi oligonucleotides directed specifically against different regions of mouse Fut8 (siFut8): MSS225401 (sense 5′-CCAGGUCUGUCGGGUUGCUUAUGAA-3′ and anti-sense 5′-UUCAUAAGCAACCCGACAGACCUGG-3′), MSS225403 (sense 5′-GAGAACUCUCCAAGAUUCUUGCAAA-3′ and anti-sense 5′-UUUGCAAGAAUCUUG GAGAGUUCUC-3′), and MSS285019 (sense 5′-GAGAUAUCAUUGGUGUGGCUGGAAA-3′ and anti-sense 5′-UUUCCAGCCACACCAAUGAUAUCUC-3′) were purchased from Invitrogen. Negative Universal Control Med#2 (#12935-112, Invitrogen) was used as a non-specific negative control (siCon).

### Quantitative real-time PCR reaction

RNA was isolated using the Quick-RNA Miniprep kit (Zymo Research, R1054). Total RNA (1 μg) was converted to cDNA using The ToolsQuant II Fast RT Kit (BioTools, KRT-BA06) according to the manufacturer’s instructions. The expression levels of Fut8 were determined using Fast SYBR™ Green Master Mix (Applied Biosystems, 4385610) on an Applied Biosystems 7500 fast real-time PCR system. Cycle threshold values were normalized to those of the housekeeping gene GAPDH. The average for three biological replicates was plotted as relative transcript abundance. The following primer sets were used: *Fut8* forward, 5′-GCTTGAACGCTTAAAACAGCA-3′, and reverse, 5′-AATGGGGCCTTCTGGTATTC-3′; *Sparc* forward, 5′-CACCTGGACTACATCGGACCAT-3′, and reverse, 5′-CTGCTTCTCAGTGAGGAGGTTG-3′; *Col1a1* forward, 5′-GACGCCATCAAGGTCTACTG-3′, and reverse, 5′-ACGGGAATCCATCGGTCA-3′; *Col1a2* forward, 5′-GGAGGGAACGGTCCACGAT, and reverse, 5′-GAGTCCGCGTATCCACAA-3′; Mouse *GAPDH* forward, 5′-TGCACCACCAACTGCTTAG-3′ and reverse, 5′-GATGCAGGGATGATGTTC-3′.

### Western blot analysis, immunoprecipitation, and lectin blot analysis

Cells were washed with PBS, lysed with 25 mM Tris–HCl (pH 8.0), 150 mM NaCl, 2 mM MgCl_2_, and 1% NP-40 containing protease inhibitors (Roche, 4693132001), and then sonicated. The cell lysates were centrifuged at 15,000×*g* for 15 min and the supernatants were collected. The concentration of total protein was determined using the Bradford protein assay (Bio-Rad, #5000006). Equal amounts of protein were analyzed by 10% SDS-PAGE and then transferred onto PVDF membranes (Millipore). Ponceau S (Sigma) and Coomassie Brilliant Blue stains allowed for visualization of total proteins. The membranes were blocked with PBS containing 1% BSA and 0.05% Tween 20 and then incubated with the indicated antibodies. After two washes, blots were incubated for 1 h with HRP-conjugated secondary antibody, corresponding to the species of the primary antibody that was used. After washing, reactive bands were visualized using the chemiluminescent substrate, HRP system (Millipore, WBKLS0100).

For immunoprecipitation, cells were homogenized in lysis buffer as described above. Samples (~ 1 mg of total protein) were incubated with indicated antibody overnight at 4 °C and then added to 20 μl of 50% (v/v) Protein A/G Magnetic Beads (Pierce, 88802) and incubated for 2 h at 4 °C with gentle rocking. After three washes with lysis buffer, precipitated complexes were solubilized by boiling in Laemmli sample buffer and fractionated by SDS-PAGE for Western blot analysis as described above. For lectin blot analysis, membranes were blocked with PBS containing 1% BSA and 0.05% Tween 20, detected with biotinylated LCA (Vector Laboratories, B-1045), and then incubated with HRP-conjugated streptavidin (Vector Laboratories, SA-5014).

### Scanning electron microscopy

Lung organoid samples were fixed in 0.1 M cacodylate buffer (pH 7.4) containing 2% glutaraldehyde overnight. Samples were post-fixed in 0.5% osmium tetroxide in 0.1 M cacodylate buffer (pH 7.4) for 2 h. Fixed samples were dehydrated in a graded ethanol series (30–100%). The samples were then dried with the use of a critical point dryer (Hitachi HCP-2), coated with gold and platinum by a sputter coater (Eiko IB-3) and observed by field emission scanning electron microscopy (Hitachi SU8220).

### Sample preparation for isobaric TMT tagging

The LSCs transfected with siRNA of siCon or siFut8 as described above were lysed using a buffer containing 5% sodium deoxycholate (SDC), 100 mM triethylammonium bicarbonate (TEAB) pH 8.5, 10 mM DTT, 1 mM PMSF, EDTA-free protease inhibitor cocktail (Roche, 4693132001), and PhosSTOP phosphatase inhibitor cocktail (Roche, 4906845001). The lysates were incubated at 100 °C for 10 min and then sonicated to shear the bulky DNA. The resulting lysates were clarified by centrifugation at 20,000×*g* for 10 min. Then the modified protocol of FASP was followed to digest the extracted proteins into the peptides suitable for MS-based proteomic analysis [16, 17]. Approximately 150 μg of protein lysate was mixed with 200 μl of 100 mM TEAB supplemented with 0.1% SDC (TEAB-SDC) in a filter unit having a cutoff limit of 30 kDa (Microcon-30, Merck Millipore) and the unit was centrifuged at 12,000×*g* for 15 min. Then 200 μl of TEAB-SDC was added and the filter unit was centrifuged again. To perform protein alkylation, 100 μl of TEAB-SDC with 50 mM iodoacetamide was added and mixed well for 1 min; afterwards, the sample was incubated without mixing for 30 min in the dark. The filter unit was centrifuged at 12,000×*g* for 10 min to remove excessive iodoacetamide. Three more washing steps of buffer exchange were performed by adding 150 μl of TEAB-SDC to the filter unit and then centrifuging at 12,000×*g* for 15 min. Then the filter unit was transferred to a new collection tube and 40 μl of TEAB-SDC with trypsin (Promega, V5111) was added to produce an enzyme to substrate ratio of 1:50 in the filter unit. The reaction solution was mixed well for 3 min and then incubated at 37 °C for 16–18 h.

The resulting peptides were collected by centrifuging the filter units at 12,000×*g* for 10 min, followed by further elution using 40 μl of 40 mM TEAB and 40 μl of 0.5 M NaCl, sequentially. The pooled eluents of peptides were acidified using trifluoroacetic acid (TFA) and the water-immiscible organic solvent, ethyl acetate, was added to extract the residual SDC in the aqueous phase [18]. The upper water-immiscible ethyl acetate layer was discarded. The aqueous phase, which contained the digested peptides, was partially dried using a SpeedVac (Thermo Fisher Scientific) to remove residual ethyl acetate and then subjected to peptide cleanup using Sep-Pak Tc18 solid-phase extraction (SPE) cartridges (Waters SKU: WAT054955). The resulting peptides of two biological batches of siCon-LSCs and siFut8-LSCs were labelled with the four isobaric reagents of TMT-6plex (TMT^6^-128, 129, 130, and 131; Thermo Scientific, 90061), respectively.

### Lectin-based affinity enrichment of core-fucosylated glycopeptides

To enrich core-fucosylated glycopeptides for MS analysis, we performed a modified protocol using the lectin affinity-based filter aided capture and elution (FACE) [19]. Aliquots (50 μg) of each TMT-labelled peptide sample were mixed and subjected to peptide cleanup using tC18 SPE cartridges to remove the excessive TMT reagents. The 200 μg of pooled TMT-labelled peptides/glycopeptides mixtures were dissolved using 100 μl of lectin binding buffer (50 mM Tris–HCl pH 7.6, 1 mM MnCl_2_, 1 mM CaCl_2_, and 0.5 M NaCl) and then transferred to the 30 kDa molecular weight cutoff filter unit. The 40 μl of LCA (5 μg/μl) and 40 μl of PSA (Pisum sativum agglutinin) (5 μg/μl) were added in the filter unit and mixed and incubation at 600 rpm for 90 min. Then the filter unit was centrifuged for 10 min to remove the lectin unbound peptides/glycopeptides. Three more of washing steps were performed by adding 200 μl of binding buffer to the filter unit and centrifuging at 12,000×*g* for 10 min. The filter unit was then transferred to a new collection tube and the elution step was started by adding 200 μl of elution buffer (20 mM Tris–HCl pH 7.6 with 200 mM α-methyl glucoside and 200 mM α-methyl mannoside) to the filter unit and mixing at 600 rpm for 20 min, followed by centrifuging the filter unit at 12,000×*g* for 10 min. The elution step was repeated two more times. The eluents were pooled and acidified using TFA and then subjected to peptide cleanup using tC18 SPE.

### Partial deglycosylation of core-fucosylated glycopeptides by Endo F3

The enriched TMT-labelled, core-fucosylated glycopeptides were dissolved in 40 μl of 50 mM sodium acetate, pH 4.5 solution and incubated with 2 μl of Endo F3 (0.3 U, QA-Bio, E-EF03) at 37 °C overnight for the partial deglycosylation that left the innermost GlcNAc and the fucose α1–6 linked to the GlcNAc (core fucose) still linked to the peptides. Then the TMT-labelled, simplified glycopeptides were desalted using tC18 SPE before LC–MS/MS analysis.

### Nanoflow LC–MS/MS and data analysis

The enriched TMT-labelled and Endo F3 simplified core-fucosylated peptides were subjected to peptide nanoflow LC–MS/MS analysis. Peptides were separated on a reverse phase capillary ACQUITY UPLC M-Class Peptide BEH C18 column (Waters, SKU: 186007484) with gradients consisting of 7–45% (80% acetonitrile, 0.1% formic acid) over 150 min at ∼300 nl/min. All MS and MS/MS spectra were acquired on an Orbitrap Fusion spectrometer (Thermo Fisher Scientific) equipped with a nanoelectrospray ion source (New Objective) and coupled to a nanoflow Ultimate 3000 (Thermo Fisher Scientific) ultrahigh pressure liquid chromatograph pump. The experiments were performed in the data-dependent acquisition mode to automatically isolate and fragment the top N multiply-charged precursors according to their intensities within the cycle time of 3 s. Intensity threshold was set to 50,000. Precursors were fragmented by high-energy collision dissociation (HCD) that was triggered using the stepped normalized collision energy (NCE) of 40 ± 8% (i.e., collision using triplicate NCE of 32%, 40%, and 48%). The raw MS and MS/MS spectra were processed by MaxQuant software (version 1.5.3.30) with an Andromeda search engine for protein and peptide identification in a target-decoy protein database. The precursor and fragment mass tolerance were set to 6 ppm and 20 ppm, respectively, and with up to two missed cleavages of trypsin digestion. Carbamidomethylation of cysteine and TMT6-plex (128, 129, 130, and 131) labelling for peptide N-terminus and lysine residues were set as fixed modifications. Oxidation of methionine, acetylation of protein N-terminals, deamidation of asparagine/glutamine, and the fucosylated GlcNAc (dHex-HexNAc) of asparagine were set as variable modifications. The false discovery rate was set to 1% for peptides, proteins, and modification sites, and the minimum peptide length allowed was seven amino acids.

### Expression and purification of recombinant SPARC protein

The human full-length SPARC and cDNA sequence with a His6 tag was cloned into the pcDNA3.1 mammalian expression vector, resulting in the pcDNA-hSPARC plasmid. The endotoxin-free expressing plasmids for transfection were prepared with the NucleoBond Xtra Maxi EF kit (Clontech, 740424). The pcDNA-hSPARC plasmid was transiently transfected into 293T-WT and 293T-Fut8KO cells via a PolyJet™ DNA transfection reagent (SignaGen Labroatories, #SL100688) according to the manufacturer’s instructions. The expressed protein was purified from cell lysates using affinity chromatography. For purification, clarified samples were loaded onto nickel resin HisTrap excel columns (Cytiva Lifescience) pre-equilibrated with binding buffer (20 mM sodium phosphate, 0.5 M NaCl, pH 7.4) using the ÄKTA pure system (Cytiva Lifescience). The flow-through fraction was collected, and the column washed with 10 column volumes (CV) of wash buffer (20 mM sodium phosphate, 0.5 M NaCl, and 10 mM imidazole; pH 7.4). Proteins were eluted using a 25 CV linear gradient going from 0 to 100% elution buffer (20 mM sodium phosphate, 500 mM NaCl, and 500 mM imidazole; pH 7.4), followed by 5 CV of 100% elution buffer. The fractions containing most of the protein were checked by SDS-PAGE. Purified His-tag SPARC proteins were applied to a HiTrap™ Desalting PD-10 column to exchange the elution buffer with HBS-P Buffer (10 mM HEPES and 150 mM NaCl; pH 7.4). In addition, SPARC molecules containing mutations at K150A, P261A, and H264A sites (SPARC-mut) were expressed in 293T-WT cells and purified as above.

### Biacore assays

The Biacore assays for SPARC-collagen interactions were performed using the Biacore X platform (GE Healthcare). Collagens were immobilized to CM5 chips using a standard amine-coupling protocol provided by the manufacturer. First, the carboxylic acid groups on CM5 chips were activated using a mixture of EDC and NHS solutions (GE Healthcare) at 25 °C for 7 min at a flow rate of 10 μl/min. Subsequently, about 10–15 μg of collagen protein dissolved in 100 μl of sodium acetate buffer (10 mM, pH 4.0) was injected into the system to form covalent bonds with the CM5 matrix. Finally, the chip was deactivated by ethanolamine (1 M, pH 8.5), resulting in 5000 to 6000 RU responses on the chip. For binding assays, HBS-P buffer was used as running buffer. In addition, a 20-s pulse of 5 mM sodium hydroxide dissolved in running buffer was used as regeneration buffer to remove the bound molecules from the chip surface.

### Modeling of SPARC protein structure

Structural modeling for SPARC and collagen interactions was performed as previously described [20]. The amino acid sequence alignments of human SPARC and the template structures (PDB 2V53 [21] and 1BMO [22]) were performed using BLAST [23]. The 3D structures of human SPARC were modeled using Modeller v9.12 [24] with the functions of the AUTOMODEL class in python scripts, including the functions for energy optimization and refinement. The Discrete Optimized Protein Energy method was used to select the best model with the lowest energy score from fifty initially generated candidates. Similarly, structural models of human SPARC in complex with collagen were generated by Modeller. The UCSF Chimera [25] program was applied for analysis of hydrogen bonds and rendering high-quality images for structural models.

### Molecular dynamics for glycosylated SPARC

Structural modeling for SPARC protein containing complex type N-glycans was performed as previously described [20]. Studies of molecular dynamics were performed using GROMACS (version 4.5.7-1) software [26] with Open MPI (version 1.5.4-2) software run on a CentOS (release 6.5) Linux system. We used the amber99sb-ildn force field for the generation of topologies for protein structures, processing of energy minimization, and simulation of molecular dynamics. Structures and topologies for complex types of glycan connected to N116 of human SPARC, with or without core-fucosylation, were generated using doGlycans packages [27]. Subsequently, the structures of glycosylated proteins were solvated with water using the TIP3P model defined in GROMACS, forming an in-solution system for simulation. Sodium ions were then added to neutralize the system according to the charges of glycosylated SPARC.

First, the molecular structures in the system were refined by an energy minimization process until the maximum force was lower than 100 kJ/mol/nm. Position restrained molecular dynamics were performed for 20 ps to equilibrate the distribution of the water molecules. Subsequently, molecular dynamics for the whole system were simulated at a temperature of 300 K, with 500 steps per ps for simulation of atomic motion. The coordinates of molecules were written to trajectory files every 2 ps for analysis of conformational changes and molecular interactions. The VMD software (https://www.ks.uiuc.edu/Research/vmd/) was used for making movies of molecular trajectories.

### Statistics

Microsoft Excel or GraphPad Prism 5 was used for statistical analysis. Statistical significance was set at *P* < 0.05, and data were plotted as means ± SEM. Two-tailed *t* tests were used when comparing two groups.

## Results

### Fut8 enzyme activity decreases upon differentiation of LSCs

**Fig. 1:**
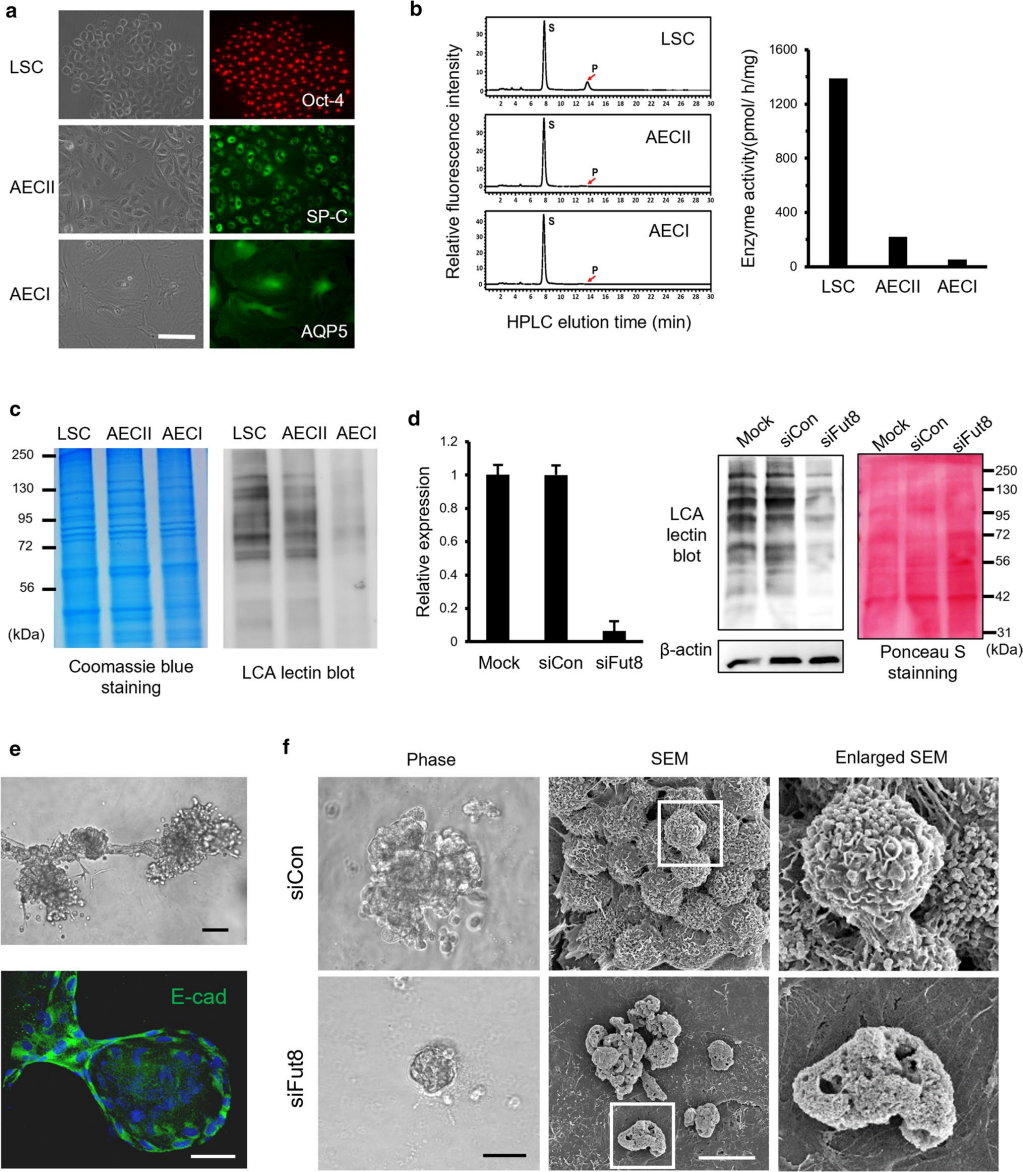
High expression of Fut8 activity in LSCs and its knockdown affecting lung organoid formation. **a** Immunofluorescent staining of LSCs, AECII cells, and AECI cells, reacted with antibodies against Oct4 (red), SP-C (green) and AQP5 (green). Scale bar, 100 μm. **b** HPLC analysis of Fut8 activity in LSCs, AECII and AECI cells as measured using a fluorescence-labeled acceptor substrate (GnGn-Asn-PAPB) in the presence of GDP-fucose. The acceptor substrate (S) and the Fut8 product (P with arrow) were eluted, respectively, at ∼8 and 14 min. The large peak at 8 min elution time represented the unreacted substrates and the peak at 14 min represented the product of Fut8 activity (left panel). Enzyme activity is shown in right panel as pmol of GDP-fucose transferred to the acceptor per hour per mg of protein. **c** Coomassie blue stained SDS-PAGE and LCA lectin blot for cell lysates of LSCs, AECII cells and AECI cells were performed, separately, to show changes of core-fucosylation (right panel). **d** qRT-PCR analysis for Fut8 expression in LSCs transfected with Fut8 siRNA (siFut8), control (siCon) or mock was performed and normalized to *GAPDH*. All data are expressed as fold-change of expression levels compared with siCon-LSCs (mean ± standard error; *n* = 3). In addition, LCA lectin blot and Ponceau S stained PVDF membrane (red) for cell lysates from these three samples were shown. The expression of β-actin was used as internal control. **e** 3D lung organoids were generated using LSCs co-cultured with endothelial cells on Matrigel. After culture for 14 days, the lung organoid showed the bronchioalveolar like structures (upper panel). Immunostaining of E-cadherin (lower panel) were visualized epithelial cells in lung organoids (green) and DAPI for nuclear counterstain (blue). Scale bars, 50 μm. **f** Phase contrast images of lung organoids generated with siCon- and siFut8-LSCs. Scale bars, 50 μm. Similarly, scanning electron microscopic (SEM) images and the enlarged SEM images from the white boxes illustrate the detailed structure of alveolar cells in lung organoids. Scale bar, 10 μm

We previously reported that the isolated CD45^−^CD54^+^CD157^+^ LSCs display the capacity for self-renewal and differentiation into alveolar epithelial cells (AECII and AECI) in a sequential manner [10, 11]. The isolated LSCs were confirmed to exhibit an epithelial cobblestone morphology and express the embryonic stem cell marker, Oct4 (Fig. 1a). After 7 days of induction, the cells became flattened, increased in size, and expressed the AECII cell marker, surfactant protein C (SP-C) (Fig. 1a). Extension of the incubation to 14 days led to further flattening and enlargement of the cells, and expression of the AECI cell marker, aquaporin 5 (AQP5), was detected (Fig. 1a). The Fut8 enzymatic activity of LSCs, AECII and AECI cells was analyzed with HPLC. Fut8 activity in LSCs was found to be 14-fold higher than in the differentiated alveolar cells (Fig. 1b). Since Lens culinaris agglutinin (LCA) lectin preferentially recognizes core-fucosylated proteins [28], the cell lysates were analyzed after SDS-PAGE with lectin blotting (Fig. 1c). While Coomassie blue staining demonstrated that the protein profiling in LSCs, AECII, and AECI cells were similar, LCA labeling signals showed significant decreases in AECII and AECI cells (Fig. 1c). These findings indicated that Fut8 activity was high in LSCs but declined markedly after differentiation.

### Fut8 silencing caused alveolar structural changes in lung organoids

It remained unclear whether core-fucosylation plays roles in LSC differentiation and alveologenesis. To investigate the effects of Fut8 activity in the development of LSCs, the small interference RNAs (siRNAs) including Fut8-siRNAs (siFut8) and a scrambled negative control (siCon) were used. These cells were shown by real-time PCR and LCA lectin blot analysis to have 80–90% inhibition of Fut8 mRNA expression and pronounced reduction of LCA binding for the core-fucosylated proteins (Fig. 1d).

Since lung is known to develop from fetal digestive tract where epithelium is enriched in the vascular stroma, we co-cultured the LSCs specifically with endothelial cells in a 3D environment to mimic their close interactions during alveologenesis. Under these conditions, these LSCs were found to form bronchioalveolar-like lung organoids as shown under phase contrast microscopy (Fig. 1e). In addition, these organoids stained with anti-E-cadherin antibody showed pores with a visible cavity inside, like the alveolar sacs of normal lung (Fig. 1e). With scanning electron microscope, lung organoids derived from LSCs displayed grape-like clusters, reminiscent of the alveolar sacs in lung. In contrast, examination with phase contrast and scanning microscope showed that the organotypic culture with siFut8-transduced LSCs exhibited dramatically changed phenotypes: the structures of alveolar sacs were collapsed and destroyed (Fig. 1f), suggesting a role of Fut8 in alveologenesis and lung development.

### Quantitative MS-based proteomic analysis for Fut8 target proteins

**Fig. 2:**
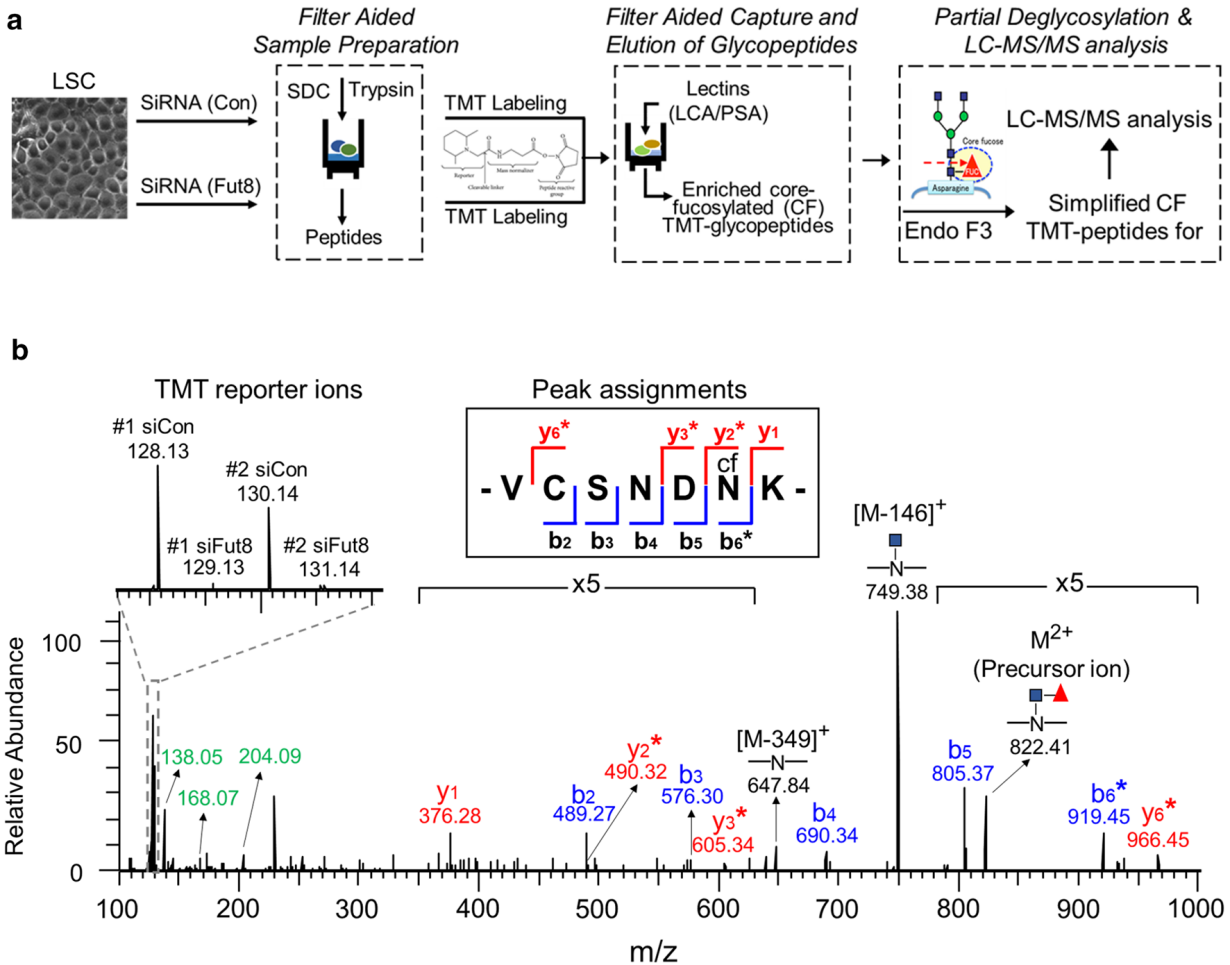
Identification of SPARC as a substrate of Fut8 using MS-based quantitative glycoproteomic analysis. **a** To explore Fut8 target proteins in LSCs, the workflow of the MS-based analysis included four major experimental approaches: filter aided sample preparation, TMT labeling, filter aided capture/elution of glycopeptides, and Endo F3 treatment/MS analysis. The detailed methodology is described in online data supplement. **b** LC–MS/MS spectral assignments of mouse SPARC N115 core-fucosylated glycopeptide. After Endo F3 treatment, M^2+^ indicates the doubly charged precursor ion (monoisotopic *m*/*z* = 822.41) attached with the innermost GlcNAc (blue square) and the fucose (red triangle) α1–6 linked to the GlcNAc. The TMT reporter ions are highlighted in the low mass range and a zoomed-in view of TMT ratios (i.e., the siFut8- vs. siCon-LSCs) is presented in the upper left region. Note that the TMT reporter ions resulted from two independent biological repeats of control and Fut8 knockdown LSCs with corresponding #1 and #2 in the zoomed-in view. There was a remarkable decline in the reporter ion signals from siFu8-treated samples. The symbol * indicates the signature loss of fucosylated GlcNAc (-349) that is labeled in b (blue) and y (red) ion assignments used to determine the peptide sequence and core-fucosylation site. The m/z of innermost GlcNAc derived oxonium ions (204.09: [HexNAc]^+^; 168.07: [HexNAc-2H_2_O]^+^, and 138.05: [HexNAc-CH_6_O_3_]^+^) are highlighted with green color. Finally, the label “ × 5” indicates that the signal intensities of MS/MS fragments in the corresponding mass range are adjusted by fivefold amplification to make the peak assignments clearer. A summary of peak assignments is shown

To systematically identify the target proteins of Fut8, we developed a quantitative MS-based glycoproteomic analysis to explore the core-fucosylated proteins in LSCs with site-specific information. As described in detail in data supplement and Fig. 2a, the isobaric tandem mass tag (TMT) labeled samples from siCon- and siFut8-LSCs were subjected to liquid chromatography-tandem mass spectrometry (LC–MS/MS) analysis. The schematic workflow consisted of four major steps: (i) preparation of tryptic peptides/glycopeptides using a modified protocol of filter aided sample preparation (FASP), (ii) TMT labeling of the resulting peptides/glycopeptides for quantitative MS comparison, (iii) affinity enrichment of core-fucosylated glycopeptides using a lectin cocktail of LCA and Pisum sativum agglutinin (PSA) over a molecular weight cutoff centrifugal filter, and (iv) partial deglycosylation of core-fucosylated glycopeptides by endoglycosidase F3 (Endo F3) to allow only the innermost GlcNAc and core-fucosylated GlcNAc were left linked to the peptides [29], and the subsequent LC–MS/MS analysis for the TMT-labeled and Endo F3 simplified core-fucosylated glycopeptides (Fig. 2a).

Following this strategy with stepped fragmentation [30], the spectrum information acquired from the dissociation with low, medium, and high collision energy, respectively, could be illustrated in a simplified core-fucosylated glycopeptide spectrum (Fig. 2b). First, the dissociation with low energy caused the fucose [mass (*m*) = 146] or fucosylated GlcNAc (*m* = 146 + 203 = 349) to dissociate from the precursor ions (M^2+^) of the Endo F3-simplified core-fucosylated glycopeptides. Thus, we could observe the corresponding signal of [M-146]^+^ for the loss of fucose and the signal of [M-349]^+^ for the loss of fucosylated GlcNAc. Second, the dissociation with medium energy resulted in serial b (blue color) and y (red color) fragment ions derived from the peptide backbone, which could be used for sequencing of the peptide. Peak assignments are also shown in Fig. 2b. Finally, the dissociation with high energy resulted in high levels of TMT reporter ions and the specific glycan oxonium ions (green color) derived from the fragmentation of the N-linked GlcNAc (Fig. 2b). Altogether, using the refinement method described above improved the efficiencies of identification and quantitation of core-fucosylated glycopeptides in LSCs.

By comparison of the relative intensities of spectra of these two samples in LC–MS/MS analysis, we observed significant downregulation at 355 specific core-fucosylation sites, which reside in 186 corresponding proteins. We then employed the following criteria to gauge the relative contributions of these apparent target proteins of Fut8: (i) a low TMT ratio of the identified glycopeptides from siFut8- vs. siCon-LSCs (Fig. 2b), (ii) the lack of changes in cDNA array after Fut8 knockdown (Table 1), and (iii) the MS/MS spectral assignments of the core-fucosylated glycopeptide (Fig. 2b). In other words, we focused particularly on those proteins which exhibited the post-translational core-fucosylation event. As summarized in Table 1, four candidates among the 186 target proteins were ranked at the top of the list; they included SPARC, LDL receptor-related protein 1, epidermal growth factor receptor, and integrin β1. These candidates all showed extremely low TMT ratios in LC–MS/MS assays and no change in cDNA array. In addition, the predicted N-glycosylated site were shown, using NetNGlyc 1.0 server, and the core-fucosylation sites from our LC–MS/MS analysis were also identified (Table 1). Moreover, the profiling of these proteins in lung tissue were examined through the Human Protein Atlas portal (Table 1).

**Table 1:** Examples of target proteins by Fut8 with altered core-fucosylation after Fut8 knockdown in LSCs

Protein Names	Gene Symbols	MS-based TMT Ratio		cDNA microarray ratio	N-glycosylation sites predicted via *NetNGlyc	Core-fucosylated sites identified by LC–MS/MS analyses	^†^ProteinAtlas in lung tissue		
		1st	2nd				RNA	Protein	IHC
SPARC	Sparc	0.04	0.07	0.88	Human: N88, N116 Mouse: N115	VCSNDNK: N115	Y	Y	Y
LRP1	Lrp1	0.05	0.05	0.88	52	17 sites	Y	Y	Y
EGFR	Egfr	0.256	0.218	0.93	15	DCVSCQNVSR: N578; TCPAGIMGENNTLVWK: N653	Y	Y	Y
Integrin β1	Itgb1	0.300	0.351	1.05	12	6 sites	Y	ND	ND

*SPARC* secreted protein acidic and rich in cysteine, *LRP1* low density lipoprotein receptor-related protein 1, *EGFR* epidermal growth factor receptor

*NetNGlyc: NetNGlyc 1.0 server (https://services.healthtech.dtu.dk/service.php?NetNGlyc-1.0)

^†^ProteinAtlas: Human Protein Atlas portal (www.proteinatlas.org)

### The matricellular protein SPARC identified as a core-fucosylated protein

SPARC in Table 1 was chosen for further studies, because this protein was reported to be a matricellular protein, which regulated not only cell–matrix interactions, but also collagen assembly [31, 32]. With NetNGlyc 1.0 server, the N-glycosylation site was predicted at a single N115 site in mouse SPARC and at two sites N88 and N116 in human SPARC. In this study, using the site-specific MS proteomic analysis, the sequence VCSNDN^cf^K of the glycopeptide of mouse SPARC, which was partial deglycosylated by Endo F3, indicate that the core-fucosylation of the N-glycan indeed occurs at N115 (Fig. 2b). In addition, the TMT reporter ions in the MS assays (Fig. 2b) and the ratios of siFut8/siCon ranging from 0.04 to 0.07 (Table 1) confirmed that the level of core-fucosylation at this site after Fut8 knockdown was significantly downregulated.

### Knockdown of Fut8 decreased the interaction between SPARC and collagen

**Fig. 3:**
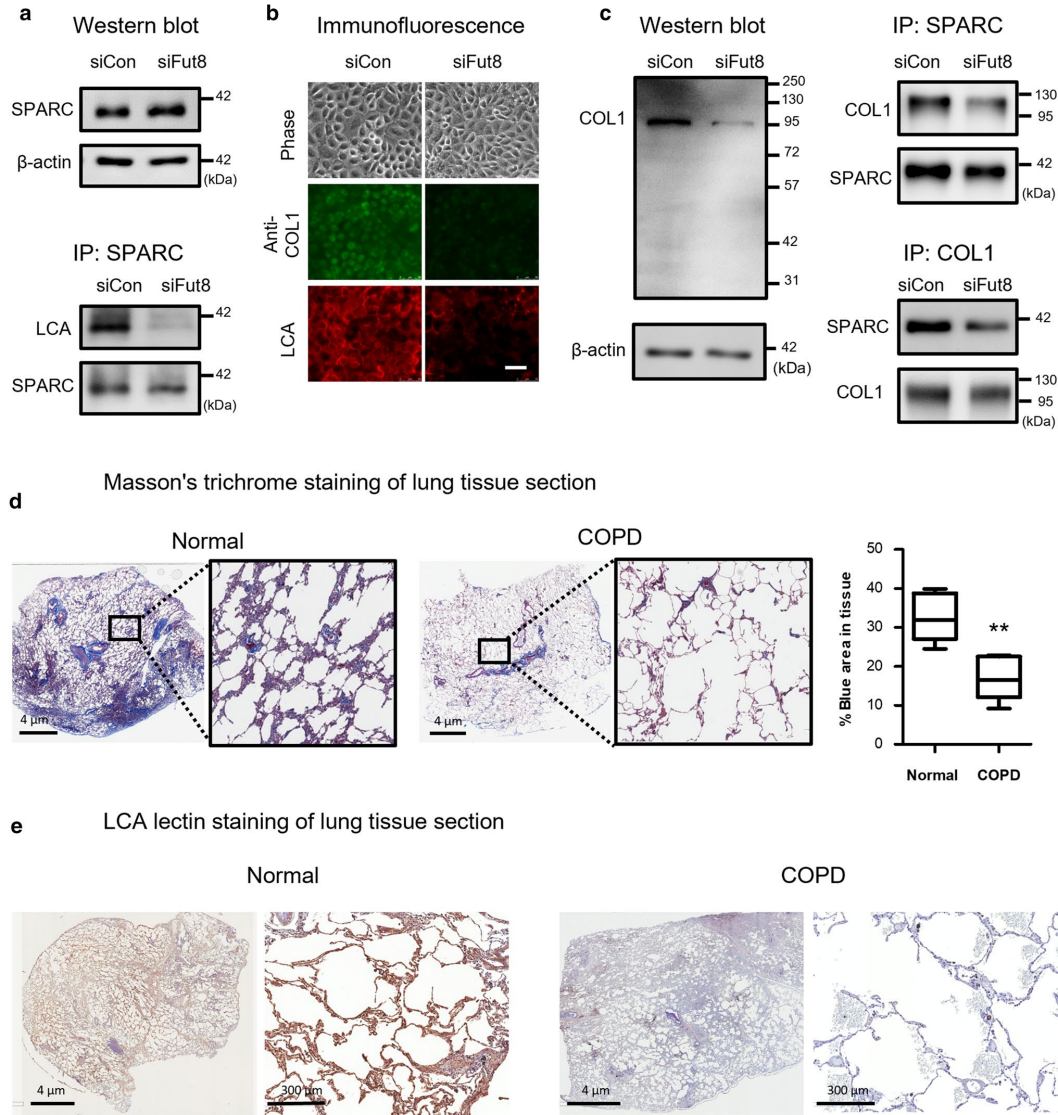
Loss of core-fucosylation in SPARC altered its binding to collagen. **a** Western blot analysis for cell lysates (left panel) and LCA lectin blot/Westin blot of anti-SPARC immunoprecipitation of lysates (right panel) from siCon- and siFut8-LSCs. β-actin was used as an internal control. **b** Phase contrast microscopy, immunofluorescence staining using anti-collagen I (COL1) antibody (green), and the LCA lectin binding of glycoproteins (red) for siCon- and siFut8-LSCs. Scale bar, 50 μm. **c** Western blot of total cell lysate from siCon and siFut8 LSCs were examined with anti-COL1 antibody using β-actin as internal control. In addition, the interaction between SPARC and COL1 was analyzed with immunoprecipitation using anti-SPARC or anti-COL1, separately. **d** Masson’s trichrome staining for detection of collagen contents (blue) in lung tissues from normal groups and those with COPD. Enlarged images in the black boxes are shown. The amount of collagen deposited (blue area) in lung tissue sections was analyzed using the software StrataQuest. Collagen composition was different in normal group (32.4 ± 2.4%, *n* = 6) and COPD group (17.2 ± 2.5%, *n* = 8). Data represent means ± standard error. **P = 0.0019, t test. **e** The representative images of LCA lectin-histochemical staining for detection of the level of core-fucosylation (brown) in normal and COPD lung sections. Scale bars, 4 μm and 300 μm, are shown

Western blots of cell lysates prepared from siCon- and siFut8-LSCs demonstrated that there was no change in SPARC expression at the protein level with Fut8 knockdown (Fig. 3a). But immunoprecipitation with anti-SPARC antibody and LCA lectin blot assays indicated a lower level of core-fucosylation in the siFut8 sample, even with the same amounts of SPARC protein. Since SPARC dictates crosstalk between cells and the ECM, such as collagen, we next assessed the effect of core-fucosylation in SPARC-collagen interaction. As shown in Fig. 3b, there was a marked reduction in collagen protein expression and a decrease in LCA binding in the siFut8-LSCs. Consistently, the expression of collagen in cell lysates from siFut8-LSCs was decreased as shown in Western blot analysis (Fig. 3c). In contrast, there was no change in mRNA expression for collagens upon Fut8 knockdown (Fig. S1a). To further confirm the decrease of interaction between SPARC and collagen complex upon Fut8 knockdown, we performed immunoprecipitation experiments using antibodies against either collagen or SPARC, respectively (Fig. 3c). As shown for immunoprecipitation of cell lysate for siFut8-LSCs, there was a significant loss of collagen I immunoprecipitated by anti-SPARC. In addition, in reciprocal experiment with anti-collagen I antibody, the amount of SPARC immunoprecipitated was also reduced in the cell lysate from siFut8-LSCs. Thus, these experiments confirmed the interaction between SPARC and collagen in LSCs.

Furthermore, immunofluorescence confocal microscopy was used to assess the SPARC and collagen expressions in lung organoids. As shown in Fig. S1b, we observed the reduction of the size of individual organoids and alterations of structure in siFut8-lung organoids. But the level of immunostaining for SPARC (red) in lung organoids was very similar in siCon- and siFut8-LSCs. On the other hand, there was significant decrease in the immunostaining for collagen I (green) in siFut8-lung organoids (Fig. S1b). Taken together, these results support the notion that the decrease of core-fucosylated SPARC impairs collagen binding, leading to the loss of collagens and structural changes in organoids. To examine whether the decrease of collagen binding is found in lung tissues of COPD patients, the amount of collagen deposited in areas of lung tissues with normal function and those with COPD was stained in Masson's trichrome assay. As shown in Fig. 3d, the blue stained collagen in COPD sections displayed a marked reduction. Moreover, with StrataQuest software analysis, the distribution of collagen deposit, measuring as blue intensity area per tissue area, was 17.2 ± 2.5% in COPD sections compared with 32.4 ± 2.4% in normal controls (*P* = 0.0019). In addition, the level of core-fucosylation was assessed with biotinylated LCA lectin. As shown in Fig. 3e, LCA positive cells in tissue sections of lung for COPD patients exhibited significantly lower LCA staining as compared to the normal lung sections, thus confirming the impaired core-fucosylation in lung tissues of COPD.

### The SPARC without core-fucosylation suppressed binding with collagen

**Fig. 4:**
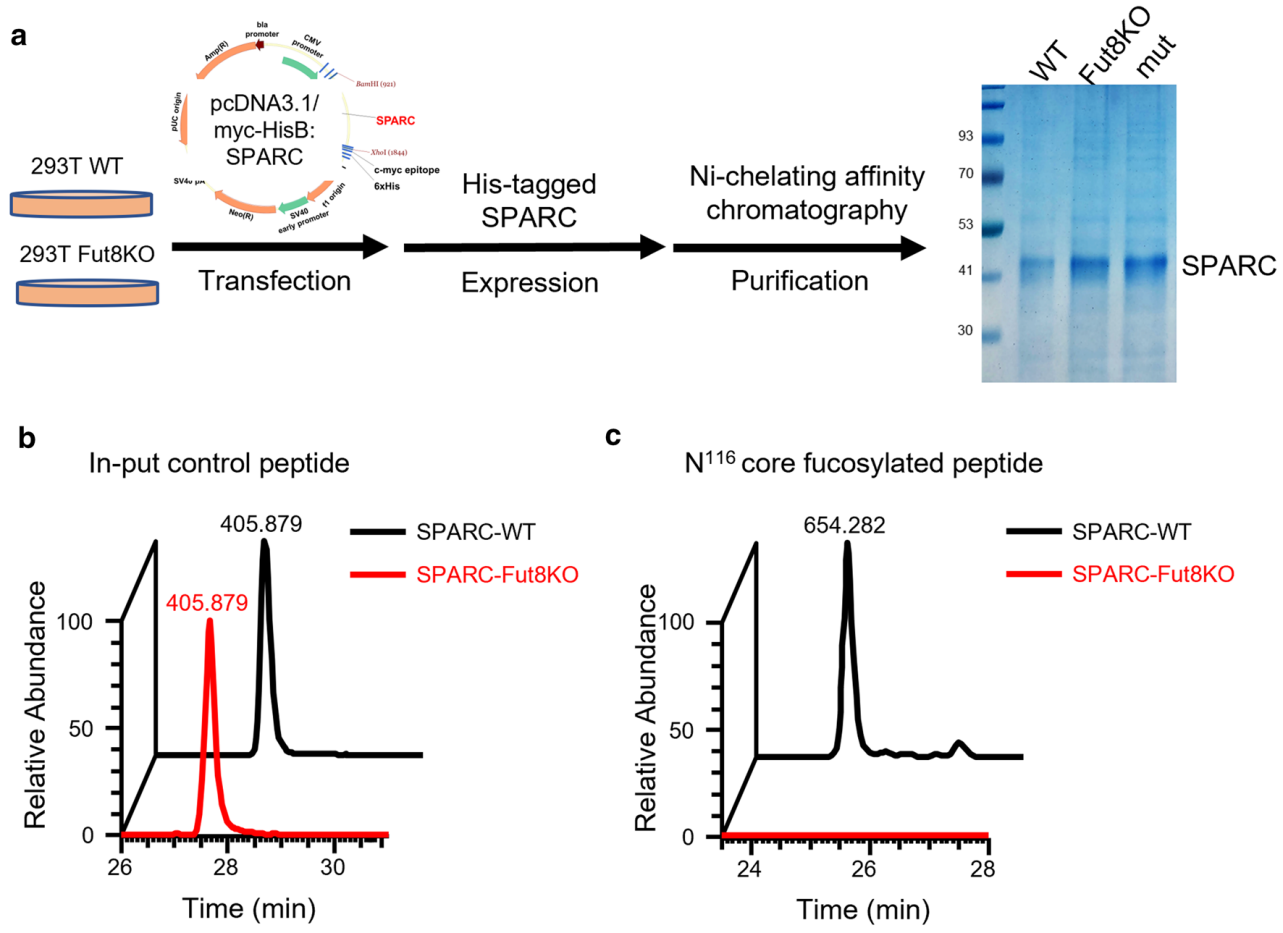
Assessment of the binding affinity of SPARC to collagen I using Biacore systems. **a** Workflow of purification of SPARC protein from 293T-WT and 293T-Fut8KO cells. These cells were transfected with pcDNA-SPARC plasmid and the His-tagged SPARC protein were purified by affinity chromatography to yield recombinant SPARC-WT, SPARC-Fut8KO and SPARC-mut (mutations at the K150A, P261A, and H264A sites) proteins. The expressed and purified SPARC proteins were subjected to 10% SDS-PAGE and stained with Coomassie blue. **b** LC–MS/MS analysis of the relative abundance of peptides derived from SPARC-WT (black) and SPARC-Fut8KO (red) purified from 293T cells. Left panel: the peak areas represented the relative levels of a SPARC derived tryptic peptide (^141^LHLDYIGPCK^150^, *m*/*z* = 405.879) without any core fucosylation modification, suggesting that the amount of in-put control from two cell samples were the same. In contrast, the peaks represented the relative levels of human SPARC N116 core-fucosylated glycopeptide (^111^VCSNDN^116cf^KTFDSSCHFFATK^129^, *m*/*z* = 654.282) that was partially deglycosylated by Endo F3 (right panel), suggesting the loss of core fucosylation in sample from SPARC-Fut8KO 293T cells (red). Note that the peak areas described above resulted from the extraction of corresponding monoisotopic *m*/*z* with 10 ppm mass tolerance

We next investigated the impact of core-fucosylation in human SPARC on its collagen binding affinity using the Biacore assay. Recombinant His-tagged human SPARC was prepared, separately, from HEK-293T (293T-WT) and FUT8-knockout HEK-293T (293T-Fut8KO) [14], and purified by Ni^2+^-based affinity chromatography to yield SPARC-WT and SPARC-Fut8KO proteins (Fig. 4a). After deglycosylation with Endo F3, the peptide fragments were analyzed by aforementioned LC–MS/MS to verify the core-fucosylated site in human SPARC. As shown left panel of Fig. 4b, the peak area of the spectra of a tryptic fragment ^141^LHLDYIGPCK^150^ from both SPARC-WT (black) and SPARC-Fut8KO (red) exhibited the similar amount of input. In contrast, the relative levels of core-fucosylated fragment, ^111^VCSNDN^116cf^KTFDSSCHFFATK^129^, of human SPARC were shown in right panel of Fig. 4b. By comparison, this core-fucosylated fragment was detected in SPARC-WT (black), but not found in the SPARC-Fut8KO (red) (Fig. 4b). Overall, these analyses confirmed that the human SPARC protein was core-fucosylated at N116, while the similar protein obtained from 293T-Fut8KO cells was not core-fucosylated. In addition, the core-fucosylation of human SPARC took place at N116 of VCSNDN^cf^K, while the similar site occurred at N115 in mouse SPARC as described above.

According to our pervious report [20], these purified human SPARCs with and without core-fucosylation were examined using Biacore assay for their binding to collagen. While recombinant SPARC-WT showed the high affinity of binding with collagen I, the binding responses (RU) for recombinant SPARC-Fut8KO, which were not core-fucosylation, were approximately 40% of the responses of SPARC-WT at 300 s (Fig. 2C of Ref. [20]). These results indicate that there was a substantial decline in the collagen binding affinity of SPARC when lacking its core-fucosylation.

### Molecular dynamics analysis of SPARC and its interaction with collagen

Human SPARC consists of three domains: a flexible N-terminal domain, a follistatin-like (FS) domain, and a C-terminal extracellular calcium-binding (EC) domain [32]. Based on X-ray crystal structure [33] and molecular modeling of SPARC [20], the collagen binding site was located at the EC domain, apparently far away from the N116 for core-fucosylation at the FS domain. To investigate how core-fucosylation of SPARC at N116 affected collagen binding affinity, the detailed fluctuations of molecular dynamics trajectories and the movies for SPARC models were now provided in the Fig. S2 and Videos 1–3.

**Fig. 5:**
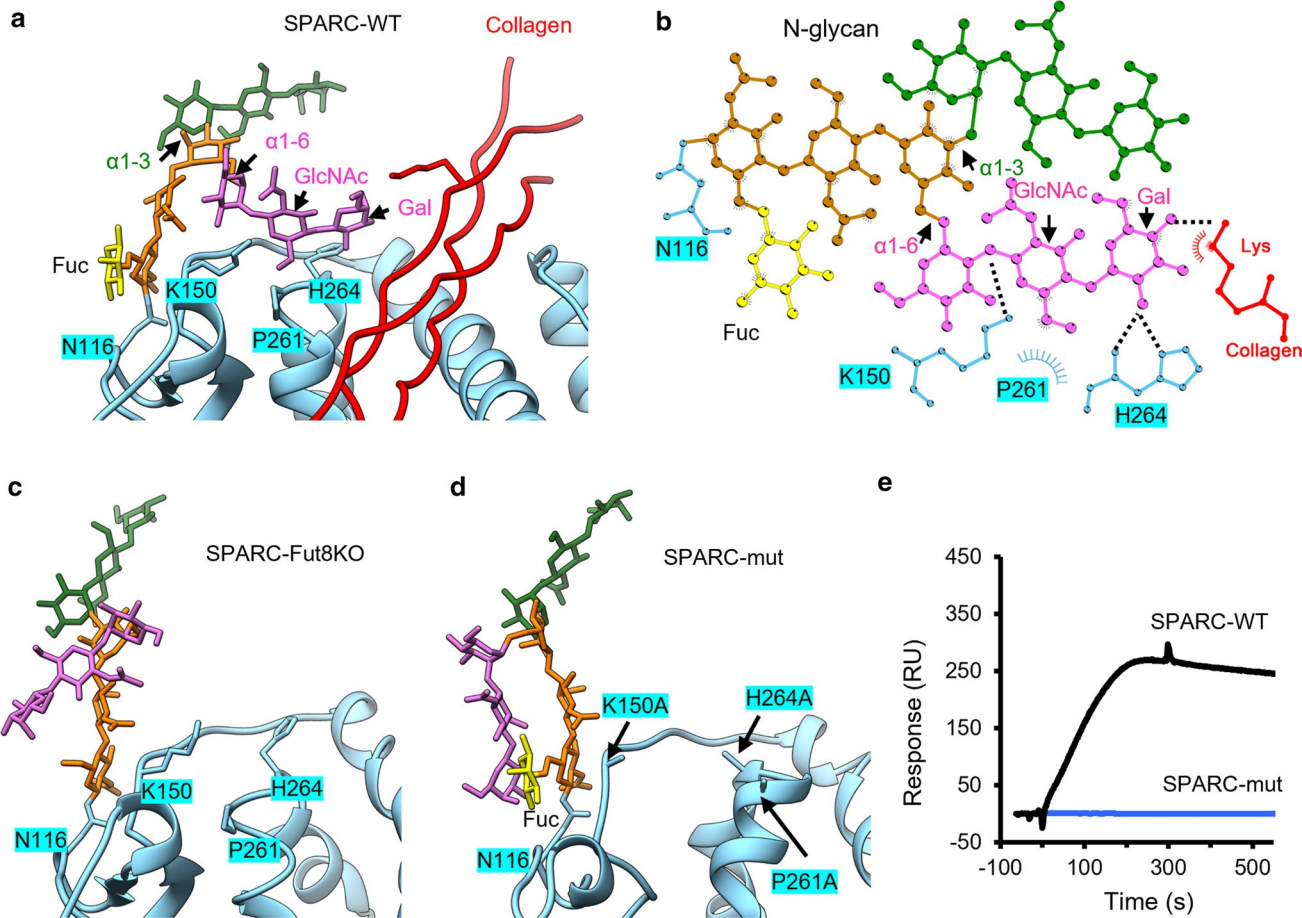
Molecular dynamics simulations revealed that core-fucosylation regulated the conformation of glycan. **a** The biantennary N-glycan of SPARC displays two arms with α1–3 (green) and α1–6 (pink) mannose (Man) to Man linkages. When the glycan at N116 of SPARC-WT with core-fucose (Fuc; yellow), the α1–6 arm (pink) was bent over to interact with amino acids of SPARC (cyan) and collagen (red). **b** The H-bond (black dashed lines) and hydrophobic interactions (radiating symbols) in the N-glycan of SPARC were represented. The GlcNAc and galactose (Gal) of the α1–6 arm formed H-bonds with K150 and H264 of SPARC, respectively; hydrophobic interaction was also observed in the area around P261. Moreover, the terminal Gal of the α1–6 arm interacted with lysine on collagen through both H-bond and hydrophobic interactions. **c** Molecular dynamics simulation for the SPARC without core-fucosylation (SPARC-Fut8KO) did not form molecular interactions with SPARC protein and collagen. **d** Molecular dynamics simulation for the SPARC with mutations at K150A, P261A, and H264A (SPARC-mut) did not display the molecular interactions that found in SPARC-WT. **e** Biacore analysis of the binding of recombinant SPARC-WT (black) and SPARC-mut (blue) to collagen I. Collagen was coupled to a CM5 sensor chip and recombinant SPARC proteins were injected into the system with HBS-P running buffer at a flow rate of 5 μl/min

As shown in Fig. 5a, structural analyses showed that the N-glycan of the FS domain in SPARC-WT displays the biantennary complex structure with α1–3 arm (green) and α1–6 arm (pink) mannose to mannose linkages. When N-glycan was attached to SPARC-WT with core-fucosylation, molecular dynamics simulation indicated that the α1–6 arm was bent over, interacting with the protein part of SPARC and the collagen (Fig. S2a and Video 1). Notably, the simulation found that the terminal galactose (Gal) and GlcNAc of α1–6 arm formed three hydrogen bonds (H-bonds) (dashed lines) with K150 and H264 residues of SPARC and also hydrophobic interactions (radiating symbol) with P261 of SPARC (Fig. 5b). Furthermore, the terminal Gal interacted with a Lys residue of collagen via one H-bond and hydrophobic interaction (Fig. 5b).

In contrast, according to molecular dynamics simulation of SPARC-Fut8KO, we found that both α1–3 and α1–6 arms of the N-glycan were freely extended with flexibility, and neither arm contacted with SPARC or collagen (Fig. 5c, Fig. S2b and Video 2), consistent with a role of core-fucosylation in the conformational regulation and molecular interactions for collagen binding.

To investigate the molecular interactions between K150, P261, and H264 of SPARC with its glycan in detail, we introduced the site-directed mutation on SPARC at K150A, P261A, and H264A sites (SPARC-mut). As shown in Fig. 5d, the features of molecular interaction in SPARC-WT were not found in SPARC-mut (Fig. S2c and Video 3). In addition, the interaction between SPARC-mut and collagen was also examined using Biacore assay. The recombinant SPARC-WT and SPARC-mut proteins purified from 293T-WT cells (Fig. 4a) were loaded onto Biacore sensor chips and the sensorgrams for collagen binding measured. As shown in Fig. 5e, the SPARC-mut protein did not bind to the collagen-coupled chip in Biacore assay (blue), as compared to SPARC-WT (black), confirming that these mutation sites were involved in the molecular interactions between SPARC and collagen.

## Discussion

The hallmarks for lung tissues of COPD are small airway narrowing and obliteration, destruction of alveolar structure, lung hyperinflation, and decline of lung elasticity [34, 35]. Chronic inflammation induced by cigarette smoking is considered to play a central role in the development of COPD, which results in progression and irreversible airflow limitation. Lung epithelium is the first barrier for the inhaled irritants to produce inflammatory chemokines, such as tumor necrosis factor (TNF)-α, interleukin (IL)-1β, IL-6, and IL-8 [36]. COPD patients show increased chemokine secretions in lungs, usually correlating with the increased number of neutrophils and macrophages [37]. In addition, many reactive oxygen species are produced, inducing oxidative stress and contributing to chronic inflammation and lung damage in COPD [38]. Furthermore, cigarette smoking was known to induce chromatin remodeling and alter the balance between histone acetylation and deacetylation, which enhanced expression of NF-κB-dependent inflammatory genes [39]. Although deleterious effects of cigarette smoking on immune reactions were shown in these studies, it is also realized that there might be other coexisting mechanisms in COPD, which include different environmental risk factors, epigenetic dysfunctions such as DNA methylation, and various post-translational modifications of histones such as methylation, acetylation, etc. [40]. However, the detailed molecular description for the underlying alveolar structural alterations from the prospective of cell matrix interactions in lung tissues is lacking.

Fut8-mediated core-fucosylation is an important post-translational modification and is related to tumor progression [41] and immune response [42], as well as cell growth and differentiation [43]. Here, we found that the Fut8 enzymatic activity and the core-fucosylated proteins are high in LSCs but decline in the differentiated alveolar cells. These LSCs, which reside at bronchoalveolar junctions, have the ability not only for self-renewal and differentiation, but also for in vivo engraftment and repair of lung tissues [10, 12]. The characteristic features of pathological findings in COPD are abnormalities in terminal bronchioles and destruction of alveolar structures based on micro-computed tomography and histology analysis [44]. In addition, previous studies showed that smoking-mediated airway basal progenitor dysfunction contributes to COPD [45]. Therefore, alteration of the stem cell niche and loss of stem cell function in lung may be associated with COPD progression. Lung organoids could be used for revealing the pathological process and investigating the regulatory mechanisms during repair derived from LSCs. In this study, silencing of Fut8 showed that 3D lung organoids display the collapsed and destroyed alveolar sac structures, which mimic the structural changes observed in COPD. These findings consistent with previous reports that Fut8^−/−^ mice showed the lung emphysema-like phenotype [2], prompting us to pursue investigation of the core-fucosylated proteins involved in alveolar structural destruction in COPD.

Currently, little is known about the Fut8 target proteins and how they contribute to COPD. Wang et al. demonstrated that the core-fucosylation of TGF-β receptor-II is crucial for TGF-β signaling in the lung [2]. Loss of core-fucosylation in TGF-β receptor-II impaired the ligand binding, leading to the down-regulated Smad2 phosphorylation and the up-regulated matrix metalloproteinases (MMPs). The overexpression of MMPs enhanced the degradation of the ECM, thus contributing to the structural collapse of lung alveoli in Fut8-null mice [2]. With site-specific LC–MS/MS analysis, we had chosen the matricellular protein, SPARC, for detailed studies.

SPARC was known to associate with the ECM and modulate the cell-ECM interactions, important for development and remodeling of ECM in injured tissues [46]. SPARC was reported to bind collagen I and other collagens like types III, IV and V [33]. But, in lung tissue, collagen type I and III are the most abundant components in the interstitial matrix, while collagen type IV is the main constituent of the basement membrane. Although the altered composition of ECM is one of the pathological features in COPD [47], studies concerning various collagens in COPD have conflicting results. On one hand, a few studies showed that the alveolar walls of emphysematous lesions contain either no change or increased collagen amounts [48, 49]. On the other hand, more studies had demonstrated a reduction of total collagens and relative decrease of collagen I over collagen III [50, 51]. In addition, fragments of collagen I, III, IV, and VI by MMPs released into systemic circulation were increased and associated with mortality of COPD [52]. In the present study, the level of collagen deposition was found to decrease significantly in the lung alveolar epithelium of COPD patients. These results agree with the reports, showing that collagen degradation was increased in the lung of patients with increased mortality [53, 54]. Our studies further suggested that conformational changes of SPARC-collagen complex regulated by core-fucosylation, conceivably, could prevent the degradation of collagen and maintain the structural and functional integrity of ECM. While SPARC acts as a collagen molecular chaperon and regulates the binding for collagen in the ECM [55], our findings provide a new paradigm for the impact of post-translational core-fucosylation of SPARC, which regulates its interactions with collagen in ECM and cell–matrix communication in COPD.

Previously, SPARC was reported to be involved in the regulation of multiple biological processes, including oncogenic and tumor suppressor properties [56]. For example, higher SPARC expression was associated with metastatic potential of melanomas, gliomas, and breast cancer [57–59]. On the other hand, SPARC expression was also associated with good prognosis of neuroblastoma, ovarian, and colorectal cancer [59]. Such discrepancy could be attributed to the abilities of SPARC either to promote or to inhibit tumor progression, dependent on cell-type, tumor staging, and various interactions between cell–matrix and tumor-stroma [60]. In the lung, SPARC that was found among the tumor-associated stroma of non-small cell lung cancer is correlated with poor prognosis [61]. The overexpression of SPARC induces epithelial-mesenchymal transition (EMT), with concomitant loss of epithelial tight junctions and E-cadherin, as well as with increase of mesenchymal markers and transcription factors, such as Slug and Snail [62, 63]. After activation of EMT, SPARC enhanced migration, invasion, and development of the tumoral vasculature network by promoting tissue remodeling, cell proliferation, and excessive ECM deposition [64]. Recently, it is reported that changes of N-glycans and their related glycosyltransferases may be involved in EMT processes [41, 65]. For example, the core-fucosylated E-cadherin regulates the accumulation of β-catenin in nucleus and activation of Src kinase in lung cancer cells, thus promoting EMT [66, 67]. In the present studies, we showed that core-fucosylation in SPARC affects the deposition of ECM and structures of alveoli, presumably leading to EMT in the lung [68]. COPD is associated with chronic inflammation of the respiratory tract and immune activation during disease exacerbations [37]. It was reported that an aberrant ECM deposition by SPARC promoted the recruitment of suppressive myeloid cells [69]. In addition, SPARC expression is associated with enhanced activation of immune and inflammatory responses [64]. Thus, SPARC may play an important role in the regulation of the interaction between ECM turnover and leukocyte recruitment leading to the alterations of lung alveolar sacs in COPD pathogenesis.

**Fig. 6:**
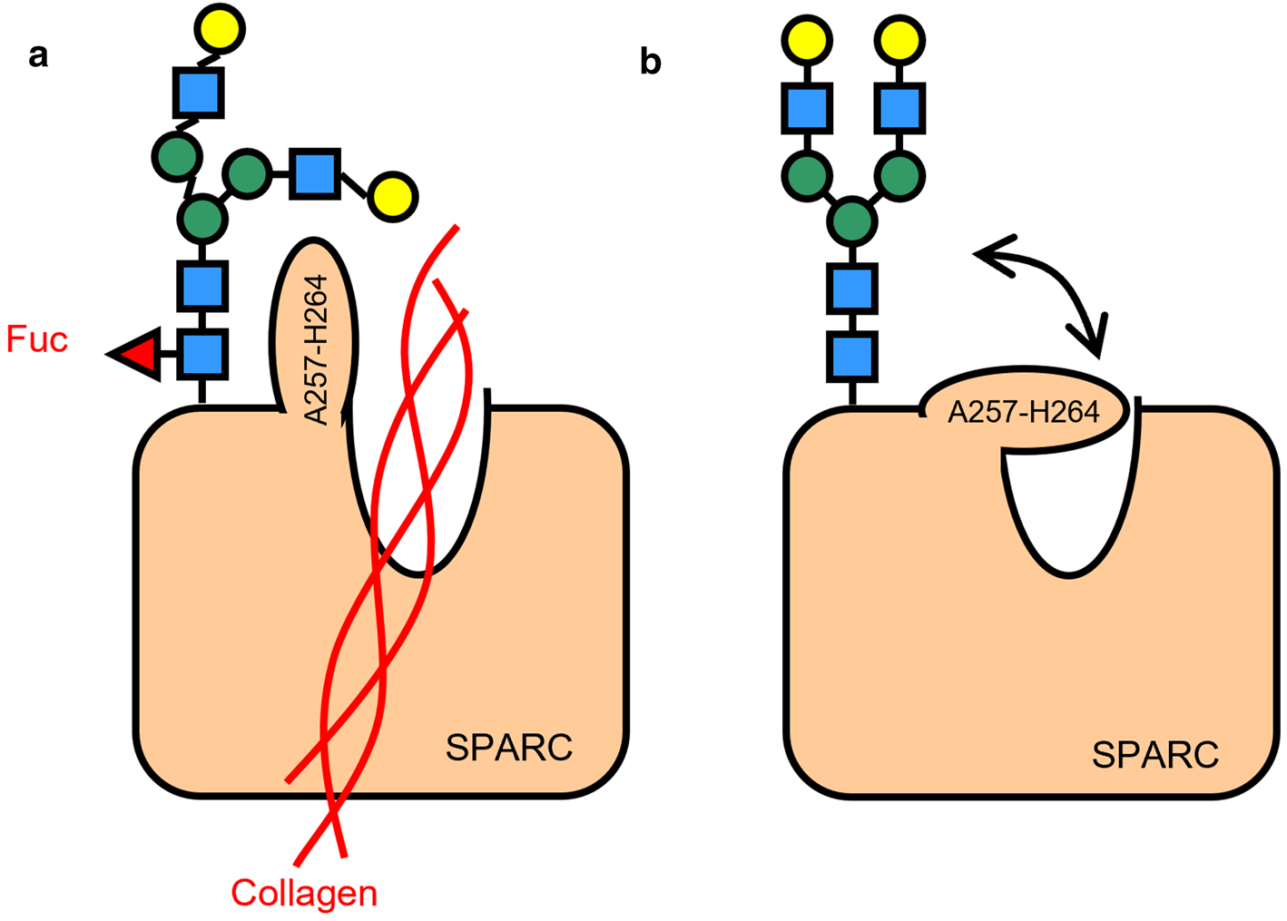
Graphical illustration of the conformational changes in SPARC and its interaction with collagen. **a** Once core-fucosylation occurred at N116 of SPARC, the core-fucosylated glycan interacted closely with the A257–H264 fragment of SPARC, keeping the SPARC conformation as an open form that enabled collagen binding. **b** When SPARC without core-fucosylation, the A257–H264 fragment of SPARC assumed conformational changes to prevent collagen from binding

In this study, we demonstrated for the first time that SPARC is a Fut8 target protein with its core-fucosylated site at VCSNDN^cf^K in both human and mouse SPARC. Recently, we have analyzed the structure of SPARC using molecular dynamics simulations based on the crystal structures of human SPARC with and without collagen binding [20–22]. When SPARC was in complex with collagen, its A257–H264 fragment folded as a helical structure, opening up the binding site for collagen [20]. In contrast, when SPARC without collagen binding, the same fragment exhibited a loop conformation shielding the collagen binding site [20]. Thus, this particular fragment serves as a switch to alter the conformation from the closed to open form allowing access of collagen for binding [20]. One explanation is that the glycan of SPARC maintains the stability of SPARC-collagen complex structure. With Biacore and molecular dynamics simulation analyses, our study provides the first evidence that loss of core-fucosylation of SPARC impaired collagen binding. In summary, the glycan with core-fucosylation of SPARC, interacts with the A257–H264 fragment, opening the access for collagen binding (Fig. 6a). On the other hand, without core-fucosylation of SPARC, the A257–H264 fragment adapted a conformation with low affinity for collagen (Fig. 6b), waiting for the obligatory conformational changes to occur. Therefore, the structural modelling of core-fucosylation in SPARC alludes not only to a protein–protein interaction, but also to a protein-glycan interaction. Taken together, impaired core-fucosylation of SPARC reduced its binding with collagen, thereby contributing to alveolar structural changes seen in COPD. Understanding the regulatory mechanisms and cellular functions underlying SPARC core-fucosylation could provide new insights for more effective management of lung injury and developing targeting therapy for clinical application.

### Supplementary Information

Below is the link to the electronic supplementary material.

Supplementary file1 (DOCX 651 KB)

Supplementary file2 (MP4 4742 KB)

Supplementary file3 (MP4 4745 KB)

Supplementary file4 (MP4 4750 KB)

## Data Availability

All data generated or analyzed during this study are included in this published article (and its supplementary information files).

## Abbreviations

AEC: Alveolar epithelial cell
AQP5: Aquaporin 5
COPD: Chronic obstructive pulmonary disease
ECM: Extracellular matrix
EMT: Epithelial–mesenchymal transition
Endo F3: Endoglycosidase F3
Fut: Fucosyltransferase
Fut8: α1,6-Fucosyltransferase
GDP-fucose: Guanosine diphosphate l-fucose
GlcNAc: Asparagine-linked N-acetylglucosamine
LCA: Lens culinaris agglutinin
LC–MS/MS: Liquid chromatography–tandem mass spectrometry
LSC: Lung stem cell
MMP: Matrix metalloproteinase
MS: Mass spectrometry
PSA: Pisum sativum agglutinin
siRNA: Small interference RNA
SPARC: Secreted protein acidic and rich in cysteine
SP-C: Surfactant protein C
