# Supplementary material for: Loss of core-fucosylation of SPARC impairs collagen binding and contributes to COPD
Source: Cell Mol Life Sci. 2022 Jun 7;79(7):348. doi: 10.1007/s00018-022-04381-4 (PMC9174126; doi:10.1007/s00018-022-04381-4)
Supplement: Supplementary file 1 — Supplementary file1 (DOCX 651 KB) [file 18_2022_4381_MOESM1_ESM.docx]

**Supplementary Material**

**Loss of core-fucosylation of SPARC impairs collagen binding and contributes to COPD**

Tsai-Jung Wu^1^, Sheng-Hung Wang^1^, Eric Sheng-Wen Chen^1^, Hsiu-Hui Tsai^1^, Yi-Chieh Chang^1^, Yi-Hsin Tseng^1^ and John Yu^1,2*^

^1^ Institute of Stem Cell & Translational Cancer Research, Chang Gung Memorial Hospital at Linkou, Taoyuan 333011, Taiwan.

^2^ Institute of Cellular and Organismic Biology, Academia Sinica, Taipei 11529, Taiwan.

^*^Correspondence to John Yu: [johnyu@gate.sinica.edu.tw](mailto:johnyu@gate.sinica.edu.tw)

**
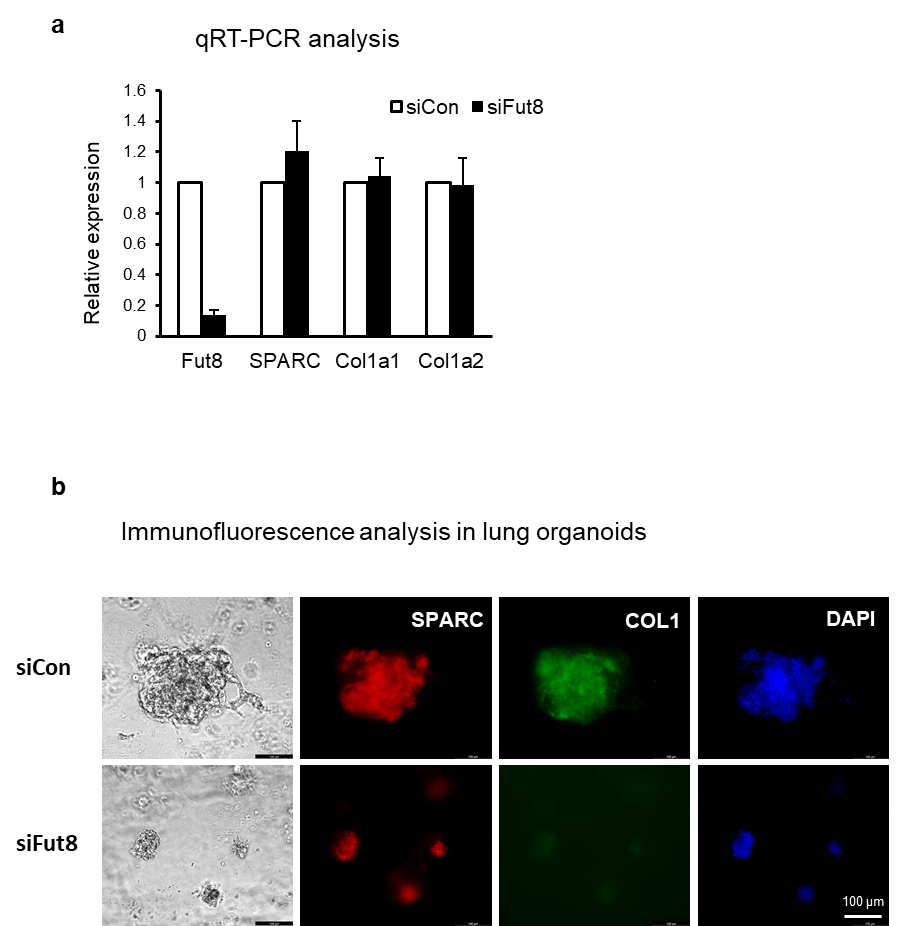
**

**Figure S1.** Effect of Fut8-knockdown on the expression levels of SPARC and collagen in LSCs and lung organoids. **a** The mRNA expression levels of *Fut8*, *SPARC*, *Col1a1* and *Col1a2* mRNA after Fut8 knockdown in LSCs were determined by quantitative real-time PCR (qRT-PCR). Relative expression is compared to siCon-LSCs after normalization with *GAPDH*. Data represent means ± standard error. **b** Representative image of lung organoids generated from siCon- or siFut8-LSCs. Confocal immunofluorescence images for the expression of SPARC (red) and collagen I (COL1; green) in lung organoids, which were counterstained with DAPI (blue). Scale bars, 100 μm.


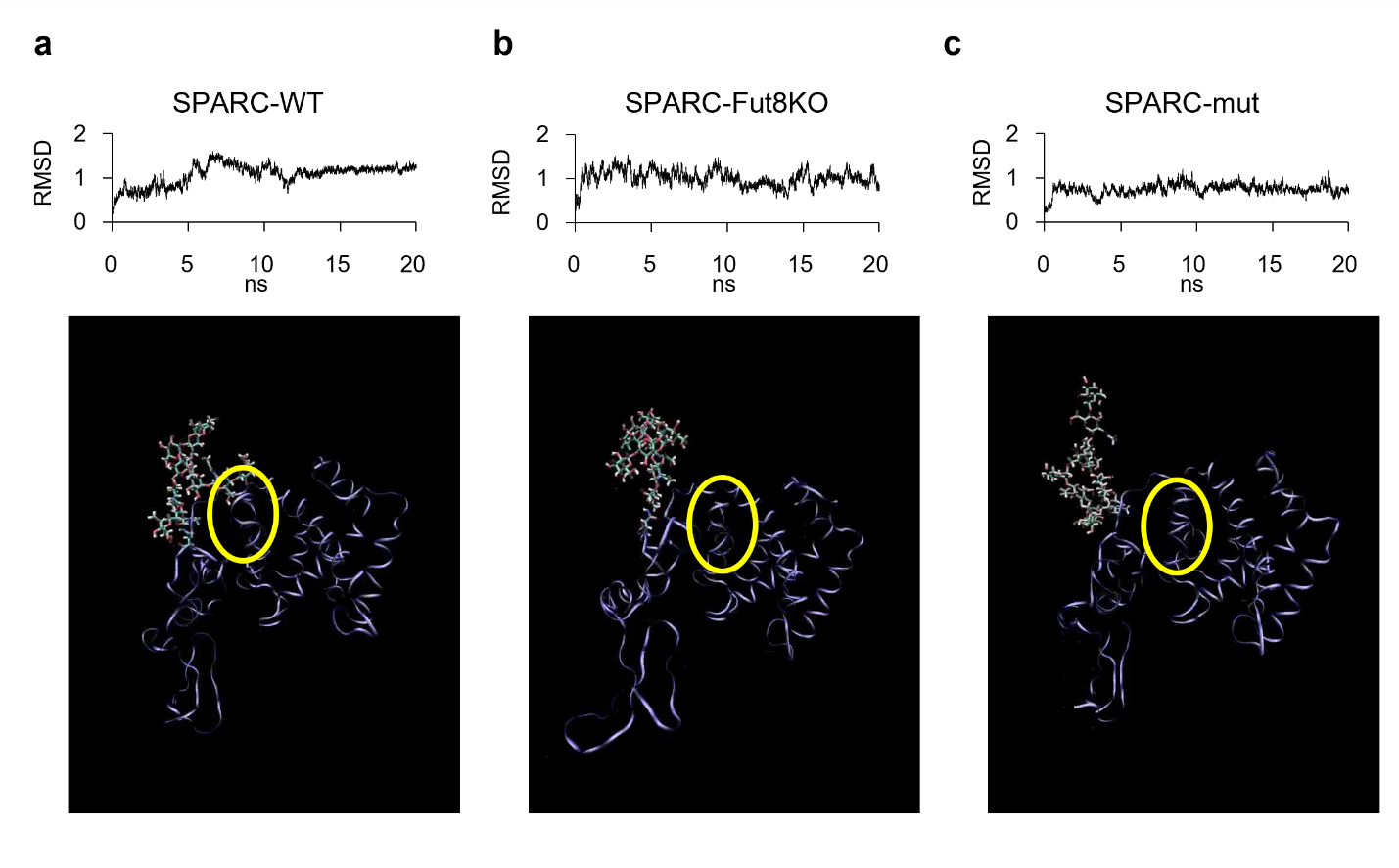


**Figure S2.** Analysis of molecular dynamics trajectories and equilibrated conformation of SPARC-WT, SPARC-Fut8KO and SPARC-mut. Upper panels show the values of root-mean-square deviation (RMSD) for the fluctuation of molecular movements. Lower panels show the equilibrated conformation of molecular dynamics simulations (MD). The yellow circles indicate the A257-H264 fragment. **a** MD for SPARC-WT with core-fucosylation. The glycan with core-fucosylation at N116 interacted with the A257-H264 fragment of SPARC. **b** MD for SPARC-Fut8KO without core fucosylation, **c** MD simulation for the SPARC with mutations at K150A, P261A, and H264A (SPARC-mut).

**Video Legends**

Video 1. **Molecular dynamics simulation of SPARC-WT.** Simulation of SPARC-WT with an N-glycan with core-fucosylation, related to Figure 5a and Figure S2a.

Video 2. **Molecular dynamics simulation of SPARC-Fut8KO.** Simulation of SPARC-Fut8KO with an N-glycan without core-fucosylation, related to Figure 5c and Figure S2b.

Video 3. **Molecular dynamics simulation of SPARC-mut.** Simulation of SPARC-mut (mutations at the K150A, P261A, and H264A sites) with an N-glycan with core-fucosylation, related to Figure 5d and Figure S2c.
